# Effect of *Nigella sativa* on reproductive system in experimental menopause rat model 

**Published:** 2016

**Authors:** Saadat Parhizkar, Latiffah Abdul Latiff, Ali Parsa

**Affiliations:** 1 *Medicinal Plants Research Centre, Yasuj University of Medical Sciences, Yasuj, Iran*; 2 *Department of Community Health, Faculty of Medicine and Health Sciences, University Putra Malaysia (UPM), Malaysia*; 3 *Department of Chemistry, College of Science, Shahre Rey Branch, Islamic Azad University, Tehran, Iran*

**Keywords:** *Menopause*, *Nigella sativa*, *Ovariectomized rats*

## Abstract

**Objective:**

Menopause is the condition when regular menstrual periods cease and may be accompanied by psychological and physical symptoms. The purpose of current study was to determine *Nigella sativa* effects on reproductive system in experimental menopause animal models.

**Materials and Methods:**

A series of experiments was conducted to investigate the effects of different dosages of *N. sativa* (first experiment), various extracts of *N. sativa* (second experiment) and some of its ingredients (third experiment) on selected menopausal parameters of ovariectomized (OVX) rats. Forty different OVX rats were equally divided into 5 groups and administered with one of the following treatments for 21 days: conjugated equine estrogen (positive control), distilled water or olive oil (negative control), treatment groups (*N. sativa*300, 600 and 1200 mg/kg in the first experiment), (300mg/kg methanol, hexane and SFE extracts of *N. sativa* in the second experiment) and (linoleic acid 50 mg/kg, gamma linolenic acid 10mg/kg, and thymoquinone 15mg/kg in the third experiment).

**Results:**

The results demonstrated that *N.sativa* exert estrogenic effect were exhibited through uterotrophic assay and vaginal cell cornification as well as blood estrogen level. Furthermore, low dose *N. sativa*, methanol extract and linoleic acid had prominent estrogenic like effects which were significantly different from those of control group (p<0.05) in different experiments.

**Conclusion:**

The finding indicated the probable beneficial role for *N. sativa* in the treatment of postmenopausal symptoms and possibility of using *N. sativa* as an alternative to hormone replacement therapy (HRT) for post menopause in human.

## Introduction

Menopause is the period in a woman’s life when hormonal changes cause menstruation to cease permanently (Andrews, 1995 ▶) and may be accompanied by psychological and physical symptoms (Bush, 2006 ▶). This is due to ovarian failure and estrogen deficiency which will influence the quality of life (Coad and Dunstall, 2005 ▶). The World Health Organization (WHO) reported that by 2030, there will be 1.2 billion postmenopausal women worldwide (Anon, 2003 ▶). Therefore, although menopause seems like a natural process, it is a period that must be definitely followed and treated (Browngoehl, 2000 ▶). The experience of menopause varies greatly from one woman to another. For some, it is completely symptom free. Others may require assistance to cope with physical and psychological effects of menopause. For women who need assistance, a range of options and supports are available such as lifestyle changes, medical treatments and complementary approaches (Bones, 2006 ▶). Hormone replacement therapy (HRT) is being used to relieve postmenopausal symptoms thereby improving the quality of life. Prolonged exposure to HRT, however, gives side effects. As such, Complementary and Alternative Medications (CAMs) is being used as an alternative to HRT. *N. sativa *(Black seed) is a spice plant which has been traditionally used for culinary and medicinal purposes (Keyhanmanesh et al., 2014 ▶). It is used to treat various illnesses and improve health status (Cheikh-Rouhou et al., 2008 ▶) and its beneficial effect on milk production was emphasized in folk medicine as well as scientific research (Hosseinzadeh et al., 2013 ▶). Furthermore, its beneficial effects in gynecologic disorders have been reported in Ayurvedic (Atul et al., 2005) and Unani medicine (Al-Jishi, 2002). Conversely, among a long list of examples which claimed usefulness of *N. sativa *in medicine, very few studies have reported its influence on the reproductive system and almost all of them have focused on the male reproductive system.There are a wide range of studies which proved its safety in animals and human (Dollah et al., 2013a ▶, 2013b ▶, Babazadeh et al, 2012 ▶). Despite of various studies on different applications of *N. sativa*, there is a few scientific studies on its application in menopausal women (Latiff et al., 2014 ▶). Thus, these experiments were conducted to determine *N. sativa* effects on reproductive system in experimental menopause rat model. The uterotrophic and vaginal cornification assays inovariectomized rats are classical methods to demonstrate estrogenic activity of chemicals or natural compounds (Clode, 2006). In this study, uterotrophic assay, morphological analyses of the uterus, vaginal cornification assay and serum estrogen level were used to study the estrogenic activity of *N. sativa*. 

## Materials and Methods

**Animals**

The protocol of the study was approved by Animal Care and Use Committee (ACUC), Faculty of Medicine and Health Sciences, University Putra Malaysia (UPM) with UPM/FPSK/PADS/BR/UUH/F01-00220 reference number for notice of approval. One hundred twenty female albino Sprague-Dawley rats weighting 250 to 350 g aged 4 months were supplied by animal house of Faculty of Medicine and Health Sciences, University Putra Malaysia. The animals were housed in a single temperature controlled (29 to 32°C) cage and 50 to 60% relative humidity with12h/12h dark/light cycle. The animals were allowed to acclimatize for at least 10 days before the start of the experiments. The rats were fed with a standard rat chow and allowed to drink water *ad libitum*. All animal received care according to the criteria outlined in the “Guide for care and use of laboratory animals” prepared by the ACUC of Faculty of Medicine and Health Sciences, University Putra Malaysia and animal handling were conducted between 08.00 and 10.00 am to minimize the effects of environmental changes. Vaginal smear was also examined daily.

**Vaginal smear**

Vaginal smears were studied to monitor cellular differentiation and to evaluate the presence of leukocytes, nucleated epithelial cells, or cornified cells. Vaginal smear samples were collected between 08.00 and 10.00 am daily. The vaginal smears were prepared by washing with 10 μl of normal saline (NaCl 0.9%) and thinly spread on a glass slide. They were allowed to dry at room temperature and then stained using Methylene blue dripping. After 30 min, the slides were rinsed in distilled water and allowed to dry. The smears were studied using the light microscope (40x) and the cell type and their relative numbers were recorded. Vaginal smear cell counts were randomly performed on 100 cells.The percentage of cornified cells was determined according to Terenius (1971) ▶ using the following formula:

$\mathrm{Percentage of Cornified Cells}=\frac{\mathrm{Cornified Cells}}{\mathrm{Cornified Cells}+\mathrm{Nucleated Cells}+\mathrm{Leucocytes}}\times 100$

**Blood collection**

The blood samples were collected at three different times, namely on day 0 (pre-treatment), day 11 (during treatment) and day 21 (after treatment). The rats were fasted for 12 hr before blood collection. Prior to blood sampling, the rats were anesthetized with diethyl ether to ease handling. The blood samples were collected by cardiac puncture using 25G, 1" needle. Approximately 2 ml of blood samples were taken and dispensed into labeled plain tubes. The blood samples were then centrifuged at 3000 rpm for 10 min to separate the sera. The serum was stored at -80°C until further use.

**Post-mortem and uterine horn histological study**

At the end of the experiment, the rats were weighed and sacrificed under chloroform anesthesia. The uterus was removed and freed from all connective tissue prior to wet weight recordings. To account for individual differences in body weight, an adjusted uterine weight was used for statistical significance calculation. Routine histological processes were employed and one horn from each rat was randomly selected and transversely cut into three equal portions known as proximal, middle and distal. Each portion was prepared as a block and from each block three ribbons were randomly chosen and examined under a light microscope (Olympus CK2) and then, measurements were carried out. For morphological analyses of the uterus, the known E2-induced features including shape and height of the luminal and glandular epithelial cells, mitotic figures, hypertrophy and hyperplasia of glands and endometrial epithelium as well as number and types of glands, presence of leucocytes and hypervascularity were recorded. The thickness of the endometrium and myometrium were also measured. Appropriate image capture was done using a light microscope (Olympus CK2) coupled to a camera (Olympus BX 41). Measurement was carried out with image analysis software (AxionVision 4.2 RELCarl Zeiss, Jena, Germany).

**Plant materials and extractions**

*N. sativa* seeds (imported from India) were purchased from a local herb store in Serdang, Malaysia. Voucher specimens of seeds were kept at the Cancer Research Laboratory of Institute of Biosciences and the seeds were identified and authenticated by Professor Dr. Nordin HjLajis, Head of Laboratory of Natural Products, Institute of Bioscience, University Putra Malaysia. The seeds were grounded to a powder shape using an electric grinder (National, Model MX-915, Kadoma, Osaka, Japan) for 6 min. Homogenized and grounded samples (100 g) were soaked overnight insolvents at a ratio of 1:5 (w/v). Two different solvents were used, namely n-hexane (Pu: 99%, Merk, Darmstadt, Germany) and methanol. The mixture of sample and solvent were covered with aluminum foil, and shacked using a shaking incubator (HeidolphUnimax 1010, Germany) at 5 to 7 rpm for 90 min. Then, solvents were filtered using Whatman paper number 1. The residues were re-soaked with fresh solvent two times to ensure the complete extraction of the oil. Solvents were completely evaporated using a rotatory evaporator (HeidolphLaborata, Germany) at 50°C and 90 rpm that yielded a blackish-brown and yellowish concentrates of methanol and hexane extract, respectively which were kept at -20°C prior to use. The extraction values (w/w %) of methanol and hexane extract were 29 and 33%, respectively. Extraction of essential oil from the seeds of *N. sativa *was also done using the speed Supercritical Fluid Extraction (SFE) instrument. The seed powder of *N. sativa *was weighed at 150 g with digital scale balance (Shimadzu Model, Japan) before placing in the extraction vessel. The oil extract was obtained at 60 MPa and 40°C by means of SFE set (SFE-1000F Thar US Technology, USA). SFE flowrates were maintained at 20.00 ml/min using a variable flow restrictor. The yellowish-brown color yield (26%w/w %) was collected within 3 hrand stored at -20°C prior to use. The collected pressure and temperature were 0.1 MPa and 25°C, respectively. The extraction was carried out with pure CO_2_.

**Experimental design**

**Figure 1:**
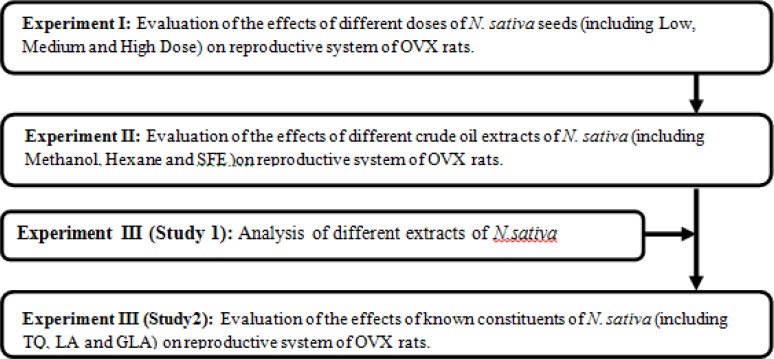
Flow diagram of the experimental studies to determine the effect of *Nigella sativa*on reproductive system of OVX rats

Three experiments were conducted to investigate the effects of *N. sativa *in OVX rats (Figure 1).Since some variables including age could affect rats condition and study results, different animals were used in these experiments. Therefore, a total of 120 OVX rats were used in three experiments in each 5 groups of eight OVX rats were used. The first experiment was to determine the effect of *N. sativa *seeds on selected menopausal parameters (estrogen levels, uterine histological changes, vaginal epithelial cell cornification) of ovariectomized rats. Prior to each experiment, rats were ovariectomized in order to induce menopause and to investigate biochemical changes following *N. sativa *supplementation. Their ovariectomy was performed during a distrous cycle to keep the consistent lowest levels of sex hormones in rats. Surgery of the animals was conducted under a combination of xylazine and ketamine (10 and75 mg/kg, i.p., respectively) anesthesia. Bilateral ovariectomy was performed through a dorso-lateral approach with a small lateral vertical skin incision (Parhizkar et al., 2008 ▶). The ovariectomized animals were acclimatized at the Animal House of Faculty of Medicine and Health Sciences for one month prior to supplementation. The seeds of *N. sativa* and commercial chow pellet was grounded evenly using an electric blender (Waring Commercial Blender, USA) to yield a fine powder. Pellet supplementations were prepared as three doses of 300 (low), 600 (medium), and 1200 (high dose) mg/kg/body weight. Forty OVX rats were equally divided into 5 groups, supplemented for 21 days and received either different dosages of *N. sativa* (as treatment groups) or CEE (0.2mg/kg) (Wyeth Montreal, Canada), or distilled water (1ml) (as positive and negative control, respectively). *N. sativa*-treated groups supplemented with chow pellet added to different doses of *N. sativa, *while CEE and distilled water were administered by intra-gastric gavage.

The second experiment was conducted to determine the effects of *N. sativa* extracts obtained by different methods of extraction on similar parameters checked in experiment one except uterine histological study. Five groups of eight OVX rats (totally 40 rats) were treated for 21 days with 300 mg/kg (which caused prominent estrogenic effect according to the result of the first experiment) of different extracts of *N. sativa* (SFE, methanol and hexane extracts), CEE (0.2mg/kg) or olive oil (1ml/day) through intra-gastric gavage. 

The third experiment (consisted of two steps) was designed to determine the fatty acid composition of *N. sativa *and investigate the effects of some of its constituents on similar parameters as in experiment two. In the first step of this experiment, various extracts of *N. sativa *were analyzed for their fatty acid composition. In step two, 40OVX rats (five groups of eight rats) were used and three different chemical constituents of *N. sativa *(linoleic acid 50 mg/kg, gamma linolenic acid 10mg/kg and thymoquinone 15mg/kg) were given to rats through intra-gastric gavage for 21 days. Doses were chosen according to the finding of the second experiment and the first step of the third experiment. CEE (0.2mg/kg) and olive oil (1ml/day) were administered to the control groups.

**Statistical analysis**

Data were expressed as means ± standard deviation and analyzed using SPSS windows program version 15 (SPSS Institute, Inc., Chicago, IL, USA). One-way Analysis of Variance (ANOVA) and General linear Model (GLM) followed by Duncan Multiple Range Test (DMRT). A p-value less than 0.05 (p<0.05) was considered to be significant.

## Results

**Figure 2:**
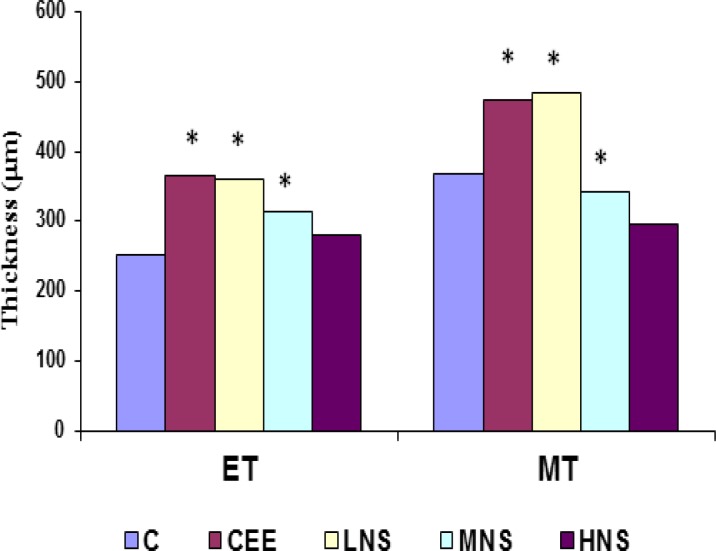
Means of Endometrial Thickness (ET) and Myometrial Thickness (MT) (µm) in Uteri of OVX rats supplemented with various doses of *Nigella sativa* or Conjugated Equine Estrogen. Treatment: C= control (1 ml distilled water); CEE= conjugated equine estrogen (0.2mg/kg); LNS= low dose of *Nigella sativa* (300mg/kg); MNS= medium dose of *Nigella sativa* (600mg/kg); HNS= high dose of *Nigella sativa* (1200mg/kg) groups. ET= Endometrial Thickness (µm) MT= Myometrial Thickness (µm). Data expressed as mean.

**Figure 3:**
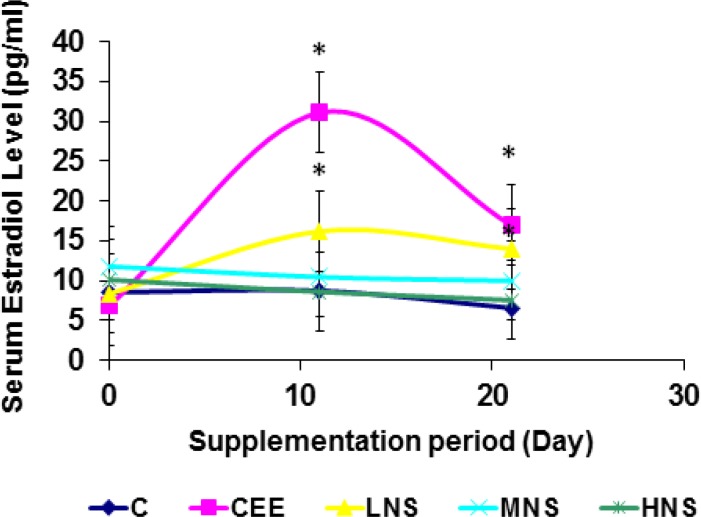
Changes of serum estradiol level (pg/ml) inOVX rats supplemented with various doses of *Nigella sativa* or Conjugated Equine Estrogen.

The results of the first experiment showed that CEE and *N. sativa* supplementation increased (p<0.05) uterine weight as compared to the controls (Table 1). These supplementations also induced histological changes in the uteri (p<0.05) similar to estrogen-induced effects (Endometrial thickness: 281±39, 365±123, 251±107 µm for NS, CEE and control, respectively) (Figure 2). Increment of serum estradiol also was observed in positive control (CEE) and low dose *N. sativa*-treated rats that were statistically different from other treatment groups (p<0.05) (Figure 3). Although there was a reduction of estradiol level after 10 days of supplementation among low dose *N. sativa* and positive control groups, but their level were significantly higher than those of control group (p<0.05). In addition, low dose of *N. sativa* supplementation was more effective in inducing the estrogenic-like effects as compared to medium and high doses. The second experiment showed that all extracts of *N. sativa* produced estrogen-like effects as compared to the control and the methanolic extract was the most effective extract (cornification of vaginal *epithelial* cell percentage 47.62%, 32.87%, 32.46% for methanol, hexane and SFE, respectively vs 62% and 0.00% for positive and negative controls, respectively) (Table 2). In the third experiment, only linoleic acid showed significant (p<0.05) estrogen-like activities (cornification of vaginal cell percentage 37.92%, 9.50%, 14.62%, 62% for LA, GLA, TQ and CEE, respectively vs 0.00% for control).

**Table 1:** Means of uterus wet and relative weight of OVX rats supplemented with various doses of *Nigella sativa* or Conjugated Equine Estrogen

**Treatment**	**Uterus Wet Weight (g)**	**Relative Uterus Wet** ** weight (mg/100g B.W)**
Control	0.19± 0.02^e^	63.49± 6.47 e
CEE	0.50± 0.01 a	174.82± 6.38 a
LNS	0.40± 0.02 b	145.93± 0.52 b
MNS	0.33± 0.01 c	112.90± 3.45 c
HNS	0.29± 0.01 d	98.91± 3.72^d^
Total	0.34± 0.01	119.21± 39.91

Data are expressed as Mean ± SD

Treatment: C: control group (treated with 1 ml distilled water); CEE: Conjugated Equine Estrogen (treated with 0.2mg/kg); LNS: Low Dose of *Nigella sativa* (treated with 300mg/kg); MNS: Medium Dose of *Nigella sativa* (treated with 600mg/kg); HNS: High Dose of *Nigella sativa* (treated with 1200mg/kg) groups.

**abcdef:** : different superscripts indicate significance at p<0.05.

**Figure 4.**
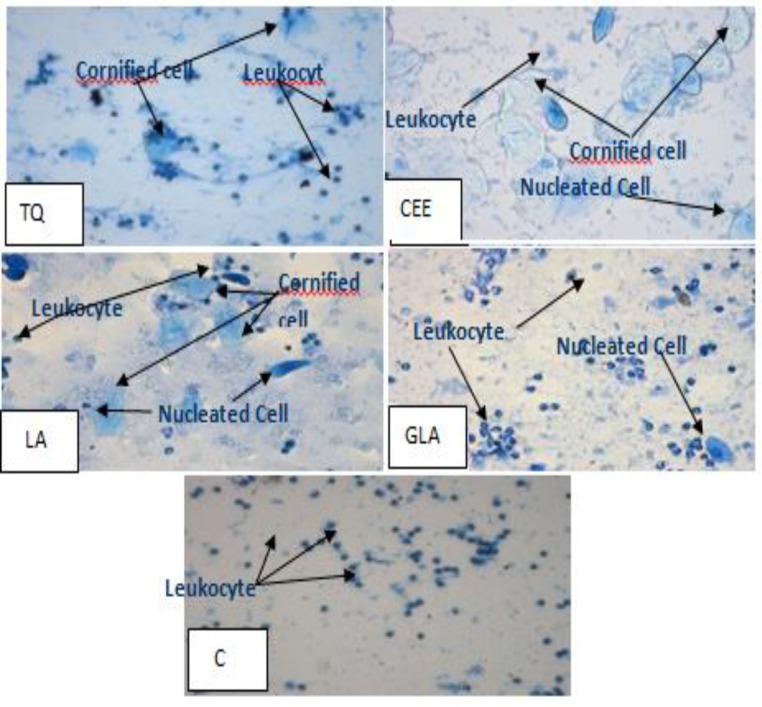
Vaginal smear of ovariectomized ratsfollowing21 days of supplementation with various ingredients of *Nigella sativa* or Conjugated Equine Estrogen. [TQ=Thymoquinone (15mg/kg/day),LA=linoleic Acid (50mg/kg/day),GLA= Gamma Linolenic Acid (10mg/kg/day),CEE= conjugated equine estrogen (0.2mg/kg/day)and C= control (1 ml Olive Oil/day)groups]. (Methylen blue staining, 40x

There was no significant difference in the percentage of cornified cells among groups at baseline and results confirmed a menopausal pattern in OVX rats. However, after treatment, cornification was observed in all treatment groups. In the first 10 days of treatment, percentage of cornified cells was increased in all groups except control group. Extending the supplementation period to 21 days consistently increased percentage of cornified cells among LA and CEE groups until the end of the treatment period which was significantly different from those of other groups (p<0.05), while cornified cells of control group remained unchanged until the end of the experiment (Figure 4).

*Significantly different from control at p<0.05.

**Table 2:** Means of vaginal cornified cell percent of OVX rats supplemented with extract from various methods of extraction of *Nigella sativa* or Conjugated Equine Estrogen

**Treatment **	**Day**		
	**0**	**11**	**21**
SFE	0.25± 0.70^c^	14.12± 22.14 c	32.46± 3.67^b^
ME	0.00± 0.00 c	14.50 ± 21.65 c	47.62± 7.96^ab^
HE	0.00± 0.00 c	12.42±20.36 c	32.87± 26.88 b
CEE	0.75± 2.12^ c^	54.16± 23.16^a^	62.00± 9.66^a^
C	0.00± 0.00 c	0.00± 0.00 c	0.00± 0.00 c

Data are expressed as Mean ± SD.

Treatment SFE= Supercritical Fluid Extract of *Nigella sativa* (300mg/kg/day); ME= Methanol Extract of *Nigella sativa* (300mg/kg/day); HE= Hexane Extract of *Nigella sativa* (300mg/kg/day); CEE= conjugated equine estrogen (0.2mg/kg/day); and C= control (1 ml Olive Oil/day) groups.

abc :different superscripts indicate significance at p<0.05.

## Discussion

The objective of this research was to determine the efficacy of *N. sativa* supplementation in improving reproductive performance and its possible effect on menopausal parameters. This research established that *N. sativa* exert estrogenic effect as shown by uterotrophic assay and vaginal cell cornification as well as blood estrogen level. An active constituent of *N. sativa*, linoleic acid also showed a weak estrogenic effect as reflected by a slight increase in blood estrogen level and vaginal cell cornification. 

These uterotrophic activities of *N. sativa* could be attributed to the flavonoid content and phenolic compounds of *N. sativa*, which has been proven to possess high estrogenic activity (Liuet al., 2004 ▶). As suggested in the previous studies, the effect of *N. sativa* powder on reproductive parameters seem to be dependent on multiple components and the synergistic action of its different constituents including nigella mine, soluble fiber, sterols, flavanoids and high content of poly-unsaturated fatty acids (Ibrahim et al., 2014 ▶). The mechanisms responsible for the effects of phytoestrogens are not clearly understood but there is suggestive evidence that phytoestrogens could act through two possible mechanisms namely, estrogen receptor-dependent and-independent (Ginsburg et al.,2000 ▶). Many studies have shown that phytoestrogens bind to estrogen receptors and show significant estrogenic effects in animals, man and cell cultures (Adlercreutzand Mazur,1997 ▶).

In this study, *N. sativa* had the desired effects on the physical, histological and biochemical parameters of the uterine tissue and vaginal cornification, thereby indicating its beneficial role in the treatment of postmenopausal symptoms. In the second study, *N. sativa* extracts displayed estrogenic activity with rising serum estradiol level and vaginal cell cornification assay particularly the methanol extract which showed more marked estrogenic effect. These results were in agreement with other studies (Haseena et al., 2015 ▶), but compared to the first study, estrogenic activity was less than crude powder. This indicates that some components removed during extraction may possibly have estrogenic action or could act as the main active component. The study also showed that the estrogenic activity of methanol extract was higher than that of hexane and SFE extracts.

The results of this study also demonstrated that the most effective extracts for exhibiting reproductive enhancement in OVX rats was the methanolic one. To investigate exact active component which is responsible for enhancement of reproductive capacity, the third study was done. Since, the role of fatty acids in reproduction is well known, in the third study, fatty acid composition of different *N. sativa *extracts which were used in the second study, were determined. 

Among essential fatty acids, linoleic acid and gamma linolenic acid were reported to display anti PMS activity (Liu et al.,2004 ▶; Ronda and Lele, 2008 ▶). Since the role of unsaturated fatty acids in improvement of reproduction performance are well known in human and animals, and considering the efficacy of *N. sativa* and its various extracts in modulating estrogenic effect in OVX rats, the third study was carried out to analyze different extracts for determining abundant fatty acids in *N. sativa.* The results illustrated that the most abundant fatty acid in all extracts was linoleic acid and its concentration in methanolic extract was higher than other extracts. Therefore, it was selected as the first active component and its estrogenic effect was investigated in OVX rats. On the other hand, gamma linolenic acid alleviatesunpleasant symptoms among postmenopausal women and its efficacy has been proven therefore,gamma linolenic Acid (GLA) waschosen as the second component for the third study. Since numerous studies also suggested the noticeable biological efficacy of thymoquinone, it was chosen as the thirdactive component. Hence, these three ingredients were investigated to observe their probable effects on menopausal symptoms. 

The results indicated that linoleic acid had a weaker estrogenic effect as compared to positive control. GLA and thymoquinone displayed the least percentage (value) of cornification and slight increase in serum estradiol level. The non-significant change in the levels of estrogen in the present study suggests that linoleic acid possibly acts directly on the estrogen receptors without enhancing the endogenous estrogen levels. *N. sativa* in powder form supplemented to chow pellet and its different extracts and linoleic acid showed estrogenic activity in OVX rats. The results of these serial studies indicated that the order of estrogenic activity was: Conjugated Equine estrogen >*N. sativa* supplemented to chow pellet >methanolic extract of *N. sativa*>hexane extract of *N. sativa*> SFE extract of *N. sativa*> linoleic acid >thymoquinone> gamma linolenic acid.
